# Limb Ischemia-Related Interventions and 30-Day Mortality After Femoro-Femoral VA-ECMO: A Pre/Post Implementation-Era Comparison

**DOI:** 10.3390/jcm15176546

**Published:** 2026-08-25

**Authors:** Robert Zilberszac, Andreas Gleiss, Bernhard Richter, Anne-Kristin Schäfer, Julia Riebandt, Patrick Haider, Thomas M. Hofbauer, Max Lenz, Georg Gelbenegger, Yalong Sun, Daniel Nöstlinger, Christian Hengstenberg, Gottfried Heinz, Walter S. Speidl

**Affiliations:** 1 Department of Cardiology, Medical University of Vienna, 1090 Vienna, Austria; bernhard.richter@meduniwien.ac.at (B.R.); thomas.hofbauer@meduniwien.ac.at (T.M.H.); max.lenz@meduniwien.ac.at (M.L.); yalongsun2001@hotmail.com (Y.S.); noestlinger.daniel@gmail.com (D.N.); christian.hengstenberg@meduniwien.ac.at (C.H.); gottfried.heinz@meduniwien.ac.at (G.H.); walter.speidl@meduniwien.ac.at (W.S.S.); 2 Institute of Clinical Biometrics, Center for Medical Data Science, Medical University of Vienna, 1090 Vienna, Austria; andreas.gleiss@meduniwien.ac.at; 3 Department of Cardiac Surgery, Medical University of Vienna, 1090 Vienna, Austria; anne-kristin.schaefer@meduniwien.ac.at (A.-K.S.); julia.riebandt@meduniwien.ac.at (J.R.); 4 Department of Clinical Pharmacology, Medical University of Vienna, 1090 Vienna, Austria; georg.gelbenegger@meduniwien.ac.at

**Keywords:** near-infrared spectroscopy, femoro-femoral, veno-arterial extracorporeal membrane oxygenation, limb ischemia, surgical amputation, distal perfusion catheter

## Abstract

**Background/Objectives**: Femoro-femoral veno-arterial extracorporeal membrane oxygenation (VA-ECMO) is associated with limb ischemic complications. Routine near-infrared spectroscopy (NIRS) monitoring and a more standardized distal perfusion strategy were introduced at our institution in 2016. We compared ischemia-related interventions, amputations, and early mortality between the treatment eras. **Methods**: Consecutive patients undergoing femoro-femoral VA-ECMO from 2012 to 2024 were analyzed retrospectively and stratified by VA-ECMO initiation before 2016 or from 2016 onward. The primary endpoint was a peripheral ischemic vascular complication requiring surgical or interventional therapy; an inclusive sensitivity definition additionally incorporated four clinically plausible but less certain events. Period-specific rates were estimated using Poisson regression adjusted for baseline distal perfusion cannula (DPC) status and extracorporeal cardiopulmonary resuscitation (eCPR). **Results**: The analytic cohort comprised 270 patients (45 pre-2016 and 225 post-2016). The primary endpoint occurred in 50 patients (18.5%, 95% confidence interval (CI) 14.1–23.7): 3/45 (6.7%, 95% CI 1.4–18.3) before 2016 and 47/225 (20.9%, 95% CI 15.8–26.8) thereafter. Adjusted rates were 4.6% (95% CI 1.0–20.5) and 18.9% (95% CI 13.6–26.3), with an adjusted risk ratio of 4.11 (95% CI 1.06–17.93). The inclusive sensitivity analysis yielded similar estimates (adjusted risk ratio 3.31, 95% CI 1.05–11.47). Five patients underwent lower-limb amputation (1/45 pre-2016 and 4/225 post-2016). Kaplan–Meier 30-day mortality estimates were 56.8% overall (95% CI 50.8–62.9), 58.9% pre-2016 (95% CI 44.9–73.5), and 56.4% post-2016 (95% CI 49.9–63.1). **Conclusions**: Ischemia-related interventions were more frequently recorded after 2016, while amputations remained rare and 30-day mortality was similar. Because monitoring, DPC practice, and other aspects of care changed concurrently, and NIRS was used without a standardized trigger algorithm, the reasons for the observed era difference cannot be determined. The findings are exploratory and do not establish causal effects of NIRS or DPC use.

## 1. Introduction

Peripheral femoral cannulation enables rapid veno-arterial extracorporeal membrane oxygenation (VA-ECMO) initiation but may compromise distal arterial flow. Acute limb ischemia is reported in approximately 17–20% of patients, with fasciotomy and amputation required in subsets of cases [1,2,3]. Large-bore arterial cannulas, peripheral arterial disease, shock-related vasoconstriction, and inadequate distal perfusion contribute to risk [1,2].

Preventive and surveillance strategies include prophylactic distal perfusion cannula (DPC) placement [4,5], serial clinical and Doppler assessment, and continuous near-infrared spectroscopy (NIRS) monitoring [6,7,8,9,10,11]. NIRS provides real-time regional tissue oxygenation data but reported intervention thresholds vary, and its effect depends on the clinical algorithm in which it is embedded [7,8,9].

In the systematic review and meta-analysis by Marbach et al., prophylactic DPC placement was associated with lower odds of limb ischemia (odds ratio (OR) 0.31, 95% confidence interval (CI) 0.21–0.47), while a small-bore (<17 French (Fr)) arterial return cannula was also associated with lower odds (OR 0.40, 95% CI 0.24–0.65) [12]. Mortality was not significantly reduced by either strategy.

From 2016 onward, our institution introduced routine lower-limb NIRS surveillance together with more frequent prophylactic DPC placement. Because these changes occurred within an evolving extracorporeal membrane oxygenation (ECMO) program, the resulting data permit an era comparison but not attribution of outcomes to NIRS alone.

The present study therefore compared rates of ischemia-related surgical or interventional limb complications, lower-limb amputation, and 30-day all-cause mortality before and after this implementation change. The analysis was designed as exploratory and hypothesis-generating.

## 2. Materials and Methods

This single-center retrospective cohort study was conducted at a tertiary academic referral center with a high-volume extracorporeal life support program. Consecutive patients undergoing femoro-femoral VA-ECMO between January 2012 and December 2024 were considered.

The year 2016 was selected a priori as the temporal cut-off because routine lower-limb NIRS monitoring was introduced alongside a shift toward more frequent, often pre-emptive DPC placement. The comparison therefore represents two treatment eras rather than a patient-level evaluation of NIRS alone.

### 2.1. Patient Population

All consecutive patients undergoing femoro-femoral VA-ECMO during the study period were screened for inclusion. Patients were excluded if they received non-femoro-femoral cannulation strategies or alternative mechanical circulatory support configurations (e.g., central cannulation, subclavian arterial cannulation, veno-venous ECMO (VV-ECMO)), as predefined in the study protocol.

The cohort was derived from an adult cardiac intensive care program. One adolescent patient (16 years) with fulminant myocarditis was managed within the same adult VA-ECMO pathway; however, this patient was excluded from the protocol-focused endpoint analyses to retain an adult-focused analytic cohort.

For patients with multiple ECMO runs during the study period, only the first VA-ECMO episode was considered.

### 2.2. Data Collection and Variable Definitions

Clinical data were extracted from electronic medical records, intensive care unit (ICU) documentation systems, and procedural reports. Variables used in the present analysis included demographics, cardiovascular risk factors, peripheral arterial disease (PAD), arrest and extracorporeal cardiopulmonary resuscitation (eCPR) status, laboratory values, NIRS ascertainment, DPC use and timing, ECMO duration, follow-up, and clinical outcomes. VA-ECMO indication, arterial cannula size, surgical versus percutaneous cannulation technique, operator experience, and validated acuity scores were not available with sufficient consistency for analysis.

Follow-up was calculated from ECMO initiation to death or the date last seen. ECMO duration was calculated from ECMO initiation to circuit discontinuation. Time to the first ischemia-related intervention was calculated in days from ECMO initiation.

### 2.3. Definition of Study Periods

Patients were assigned to the pre-2016 cohort when ECMO was initiated before 1 January 2016 and to the post-2016 cohort when ECMO was initiated on or after that date.

### 2.4. Exposure Variables

Near-infrared spectroscopy and distal perfusion cannulation.

NIRS was used as an adjunct to clinical assessment. There were no predefined saturation thresholds or a standardized NIRS-triggered intervention algorithm. Patient-level NIRS use was classified as yes, no, or not reliably ascertainable.

DPC placement was adjudicated from procedure timing and source documentation as prophylactic (placed during initial cannulation before documented hypoperfusion), reactive (placed because of suspected or documented limb hypoperfusion), or uncertain. Reactive DPC placement was considered part of the primary endpoint when performed to treat suspected or documented ischemia. For baseline adjustment, reactive DPC placement was grouped with no prophylactic DPC because it occurred after baseline. Uncertain cases were retained as a separate category.

### 2.5. Outcome Definitions

#### 2.5.1. Limb Ischemic Complications

The primary endpoint was a peripheral ischemic vascular complication requiring surgical or interventional therapy from ECMO initiation through discharge or death during the index hospitalization. Ischemia was identified from documented clinical hypoperfusion or limb ischemia that prompted an invasive intervention. Included procedures comprised reactive DPC placement or revision, fasciotomy, thrombectomy or thromboendarterectomy, percutaneous or surgical vascular reconstruction, ECMO cannulation revision for threatened limb perfusion, and lower-limb amputation. Side, indication, procedure type, and timing were adjudicated from procedural reports and the clinical record. Interventions for bleeding or independent vascular disease without documented limb hypoperfusion were excluded.

The strict endpoint required a clearly documented ischemic indication. An inclusive sensitivity analysis added four clinically plausible interventions for which the ischemic attribution was less certain. Lower-limb amputation was assessed separately as a secondary endpoint. First-event components were assigned to mutually exclusive categories using the hierarchy amputation, fasciotomy, DPC-related procedure, other vascular intervention or reconstruction, and isolated ECMO cannulation revision/other.

#### 2.5.2. Mortality

Thirty-day all-cause mortality was estimated from ECMO initiation using the Kaplan–Meier method. Patients without a documented death were censored at the date last seen.

### 2.6. Baseline Variables

Baseline characteristics included age, sex, cardiovascular risk factors, coronary artery disease, prior coronary artery bypass grafting, peripheral arterial disease, cardiopulmonary resuscitation (CPR) and eCPR status, peak lactate within 24 h, NIRS ascertainment, baseline DPC category, and ECMO duration.

### 2.7. Statistical Analysis

Continuous variables are reported as median with interquartile range (IQR) and compared between periods using Wilcoxon’s rank-sum test. Categorical variables are presented as counts and percentages and compared using Chi-square or Fisher’s exact tests, as appropriate. Analyses used available cases; the extent of missing or uncertain data is reported in the Results and table footnotes.

For the strict primary endpoint, the inclusive sensitivity endpoint, and lower-limb amputation, proportions were calculated overall and by treatment era with exact Clopper–Pearson 95% confidence intervals.

The approved study protocol prespecified exploratory Poisson regression comparing study periods with adjustment for the presence of an antegrade DPC to obtain adjusted era-specific rates. For the revised analysis, DPC exposure was refined to distinguish baseline prophylactic from later reactive placement, and eCPR was added as a further baseline covariate. The final model included treatment era, eCPR, and a three-level baseline DPC variable: no prophylactic DPC (including later reactive placement), prophylactic DPC, and uncertain status. The direct era risk ratio was derived from this model. Owing to only five amputations, no adjusted amputation model was fitted.

The primary model used the strict endpoint. The same model was repeated using the inclusive sensitivity definition. Thirty-day mortality was estimated using the Kaplan–Meier method, censoring patients still alive at the date last seen, and is reported with 95% confidence intervals. No formal post hoc power calculation or competing-risk model was performed because the analysis was exploratory and event counts were limited.

### 2.8. Statistical Software

Statistical analyses were performed using SAS 9.4 (SAS Institute Inc., Cary, NC, USA). A two-sided significance level of 0.05 was used where applicable. The primary emphasis was on estimated rates and confidence intervals.

## 3. Results

### 3.1. Study Population, Data Completeness, and Cohort Definition

Between January 2012 and December 2024, 271 consecutive femoro-femoral VA-ECMO patients met the configuration criteria. One 16-year-old patient was excluded from protocol-focused adult endpoint analyses, leaving 270 patients.

The adult analytic cohort comprised 45 patients treated before 2016 and 225 treated from 2016 onward. Age, sex, peripheral arterial disease, eCPR status, ECMO duration, endpoint status, and follow-up were complete. Peak lactate within 24 h was missing in 7/270 patients. NIRS use was not reliably ascertainable in 5/270 patients, and DPC timing/indication remained uncertain in 45/270 patients; these were retained as explicit categories rather than imputed.

### 3.2. Baseline Characteristics

Baseline characteristics by treatment era are summarized in Table 1.

Patients treated in the post-2016 cohort were significantly older than those treated before 2016. Median age was 51.8 years (interquartile range [IQR] 41.0–60.5) in the pre-2016 cohort and 57.5 years (IQR 48.1–64.5) in the post-2016 cohort (*p* = 0.010). Sex distribution did not differ significantly between eras. Male sex accounted for 66.7% of patients in the pre-2016 cohort and 74.7% in the post-2016 cohort (*p* = 0.268).

Cardiovascular comorbidities were broadly similar. Peripheral arterial disease was documented in 1/45 (2.2%) pre-2016 and 10/225 (4.4%) post-2016 patients (*p* = 0.697).

Prior coronary artery bypass grafting was more common pre-2016 (8.9% vs. 2.2%, *p* = 0.023). CPR before ECMO was frequent in both eras (62.2% vs. 65.8%, *p* = 0.648), as was eCPR (46.7% vs. 51.6%, *p* = 0.549).

Peak lactate within 24 h was similar between eras. Median ECMO duration was 4 days (IQR 2–8) in both groups (*p* = 0.521).

NIRS monitoring was documented in 9/45 (20.0%) pre-2016 and 195/225 (86.7%) post-2016 patients (*p* < 0.001). Prophylactic DPC use increased from 5/45 (11.1%) to 102/225 (45.3%); reactive DPC placement occurred in 1/45 (2.2%) and 20/225 (8.9%), while classification remained uncertain in 3/45 (6.7%) and 42/225 (18.7%), respectively (*p* < 0.001).

### 3.3. Protocol-Specified Primary Endpoint

The strict primary endpoint occurred in 50/270 patients (18.5%, 95% CI 14.1–23.7): 3/45 (6.7%, 95% CI 1.4–18.3) pre-2016 and 47/225 (20.9%, 95% CI 15.8–26.8) post-2016. Adjusted era-specific rates were 4.6% (95% CI 1.0–20.5) and 18.9% (95% CI 13.6–26.3), respectively; the adjusted post- versus pre-2016 risk ratio was 4.11 (95% CI 1.06–17.93) (Table 2).

The inclusive sensitivity endpoint occurred in 54/270 patients: 4/45 pre-2016 and 50/225 post-2016. Adjusted rates were 6.3% (95% CI 1.8–22.3) and 21.0% (95% CI 15.7–28.0), with an adjusted risk ratio of 3.31 (95% CI 1.05–11.47) (Table 3). Median time from ECMO initiation to the first strict endpoint was 3 days (IQR 2–3) among three pre-2016 events and 1 day (IQR 0–4) among 47 post-2016 events.

Among the 50 strict first events, 19 were DPC-related (all post-2016), 10 were fasciotomies without amputation (1 pre-2016 and 9 post-2016), 5 included amputation (1 and 4), 15 involved thrombectomy, thromboendarterectomy, or another vascular reconstruction (1 and 14), and 1 post-2016 event consisted of isolated ECMO cannulation revision/other intervention.

### 3.4. Lower Limb Amputation

Lower-limb amputation occurred in 5/270 patients (1.9%, 95% CI 0.6–4.3): 1/45 pre-2016 (2.2%, 95% CI 0.1–11.8) and 4/225 post-2016 (1.8%, 95% CI 0.5–4.5) (Table 4).

Because only five amputations occurred, adjusted estimates were not calculated.

### 3.5. Thirty-Day All-Cause Mortality

Kaplan–Meier estimated 30-day all-cause mortality at 56.8% overall (95% CI 50.8–62.9), 58.9% pre-2016 (95% CI 44.9–73.5), and 56.4% post-2016 (95% CI 49.9–63.1) (Table 5).

## 4. Discussion

In this single-center cohort study of femoro-femoral VA-ECMO patients, we evaluated ischemia-related limb interventions, lower limb amputations, and 30-day mortality before and after the 2016 implementation change. Ischemia-related procedures were more frequently recorded after 2016, whereas lower limb amputations remained rare and 30-day mortality was similar. The strict and inclusive endpoint analyses yielded concordant results.

The higher rate of ischemia-related interventions observed after 2016 warrants careful interpretation. Enhanced surveillance through routine NIRS monitoring may have increased detection of subclinical or early ischemic changes that would have gone unrecognized in earlier eras and may have lowered the threshold for intervention. Nineteen of 47 post-2016 first events were DPC-related, whereas none of the three pre-2016 first events were. The composite is therefore sensitive to management intensity and does not directly measure physiological ischemia severity. The later cohort was older, and changes in case mix or care may have contributed.

The present data suggest a nuanced interpretation of the relationship between monitoring strategies, limb perfusion management, and patient outcomes. Documentation systems and endpoint coding rules did not intentionally change across eras. Nevertheless, ascertainment likely changed when continuous NIRS data became available, and limb perfusion received greater clinical attention. Retrospective records cannot quantify this detection effect.

Distal perfusion cannulas were increasingly placed as part of routine practice in the later era and should be interpreted as part of an era-related management strategy rather than as an isolated patient-level risk marker. Prophylactic placement is a baseline choice; reactive placement follows hypoperfusion and may satisfy the endpoint. For adjustment, reactive placement was grouped with no prophylactic DPC, and uncertain cases formed a separate category. The DPC-adjusted Poisson framework was prespecified, but the refined classification and eCPR covariate were introduced during revision. The analysis remains exploratory and does not estimate a causal DPC effect.

The role of NIRS monitoring itself deserves particular attention. At our institution, NIRS was used without predefined intervention thresholds or a standardized NIRS-triggered algorithm. Accordingly, threshold-based adherence or the impact of specific NIRS-triggered interventions could not be assessed. The observed era differences therefore cannot be attributed to NIRS per se.

Thirty-day mortality was similar across eras, consistent with limb perfusion strategies primarily addressing morbidity rather than survival. ECMO duration was identical across eras, but exposure time varied. Patients who died early had less opportunity to develop a limb event, whereas prolonged support increased time at risk. Sparse events precluded stable competing-risk analysis. Death remains an important competing event.

The 45-patient pre-2016 cohort was substantially smaller than the 225-patient post-2016 cohort. Consequently, confidence intervals are much wider for pre-2016 estimates, especially for rare outcomes, and apparently large relative differences remain imprecise. We did not perform a post hoc power calculation because it would add no information beyond the observed estimates and confidence intervals. All estimates remain exploratory.

Several limitations should be acknowledged. The retrospective, single-center design introduces potential residual confounding and limits generalizability. The pre/post comparison captures time-related changes beyond NIRS implementation in technology, ICU care, team experience, and patient selection [13]. Arterial cannula size, VA-ECMO indication, cannulation technique, operator experience, and acuity scores were unavailable consistently. PAD occurred in only 11 patients and was not adjusted for; age had little influence and was not retained. Peak lactate was missing in seven, NIRS use was uncertain in five, and DPC classification was uncertain in 45. The composite endpoint reflects interventions and may therefore be influenced by surveillance and treatment thresholds. Residual confounding therefore remains.

Prospective multicenter studies should evaluate standardized bilateral limb-NIRS thresholds linked to predefined confirmatory testing and intervention algorithms. Such studies should distinguish prophylactic from reactive DPC placement, incorporate competing death and time at risk, and include patient-centered outcomes such as amputation-free survival and limb function.

## 5. Conclusions

In this single-center pre/post implementation-era comparison, ischemia-related interventions were more frequently recorded after 2016, while amputations remained rare and 30-day mortality was similar. The reasons for the higher intervention rate cannot be determined from these data. Concurrent changes in surveillance and DPC practice, the absence of a standardized NIRS-triggered algorithm, and residual confounding preclude causal conclusions regarding NIRS or DPC use.

## Figures and Tables

**Table 1:** Baseline characteristics by study era.

Variable	Pre-2016 (n = 45)	Post-2016 (n = 225)	*p*-Value
Age, years	51.8 (41.0–60.5)	57.5 (48.1–64.5)	0.010
Male sex	30 (66.7%)	168 (74.7%)	0.268
Hypertension	14 (31.1%)	100 (44.4%)	0.098
Diabetes mellitus	9 (20.0%)	56 (24.9%)	0.484
Coronary artery disease	10 (22.2%)	51 (22.7%)	1.000
Prior CABG	4 (8.9%)	5 (2.2%)	0.023
CPR before ECMO	28 (62.2%)	148 (65.8%)	0.648
Peripheral arterial disease	1 (2.2%)	10 (4.4%)	0.697
NIRS ascertainment	No 33 (73.3%)	No 28 (12.4%)	<0.001
	Yes 9 (20.0%)	Yes 195 (86.7%)	
	Uncertain 3 (6.7%)	Uncertain 2 (0.9%)	
ECMO duration, days	4 (2–8)	4 (2–8)	0.521
Peak lactate 24 h (mmol/L)	5.4 (1.9–14.0)	7.5 (3.5–11.6)	0.201
DPC classification	No DPC 36 (80.0%)	No DPC 61 (27.1%)	<0.001
	Prophylactic 5 (11.1%)	Prophylactic 102 (45.3%)	
	Reactive 1 (2.2%)	Reactive 20 (8.9%)	
	Uncertain 3 (6.7%)	Uncertain 42 (18.7%)	
eCPR	21 (46.7%)	116 (51.6%)	0.549

Values are median (interquartile range) or n (%). Peak lactate was missing in 7 patients. NIRS “uncertain” indicates that patient-level use could not be reliably ascertained; DPC “uncertain” indicates that timing or indication could not be reconstructed. Abbreviations: CABG, coronary artery bypass grafting; CPR, cardiopulmonary resuscitation; DPC, distal perfusion cannula; eCPR, extracorporeal cardiopulmonary resuscitation; ECMO, extracorporeal membrane oxygenation; IQR, interquartile range; NIRS, near-infrared spectroscopy; PAD, peripheral arterial disease.

**Table 2:** Strict primary endpoint: ischemia-related surgical or interventional therapy.

Group	Events/N	Rate, %	95% CI	Adjusted Rate, %	Poisson-Adjusted 95% CI
Overall cohort	50/270	18.5	14.1–23.7	-	-
Pre-2016	3/45	6.7	1.4–18.3	4.6	1.0–20.5
Post-2016	47/225	20.9	15.8–26.8	18.9	13.6–26.3

Unadjusted proportions are shown with exact Clopper–Pearson 95% confidence intervals. Poisson-adjusted rates and the risk ratio account for treatment era, baseline DPC category (no prophylactic DPC, prophylactic DPC, uncertain), and eCPR. Adjusted post- versus pre-2016 risk ratio: 4.11 (95% CI 1.06–17.93). Abbreviations: CI, confidence interval; DPC, distal perfusion cannula; eCPR, extracorporeal cardiopulmonary resuscitation.

**Table 3:** Sensitivity analysis: inclusive ischemia-related intervention endpoint.

Group	Events/N	Rate, %	95% CI	Adjusted Rate, %	Poisson-Adjusted 95% CI
Overall cohort	54/270	20.0	15.4–25.3	-	-
Pre-2016	4/45	8.9	2.5–21.2	6.3	1.8–22.3
Post-2016	50/225	22.2	17.0–28.2	21.0	15.7–28.0

The inclusive endpoint additionally counted four clinically plausible but less certain ischemia-related events. Poisson-adjusted rates use the same covariates as the strict model. Adjusted post- versus pre-2016 risk ratio: 3.31 (95% CI 1.05–11.47). Abbreviations: CI, confidence interval; DPC, distal perfusion cannula; eCPR, extracorporeal cardiopulmonary resuscitation.

**Table 4:** Secondary endpoint: lower-limb amputation.

Group	Events/N	Rate, %	95% CI	Adjusted Rate, %	Adjusted 95% CI
Overall cohort	5/270	1.9	0.6–4.3	Not estimated	Not estimated
Pre-2016	1/45	2.2	0.1–11.8	Not estimated	Not estimated
Post-2016	4/225	1.8	0.5–4.5	Not estimated	Not estimated

Proportions are shown with exact Clopper–Pearson 95% confidence intervals. Adjustment was not performed because the number of events provided insufficient degrees of freedom. Abbreviation: CI, confidence interval.

**Table 5:** Secondary endpoint: 30-day all-cause mortality after extracorporeal membrane oxygenation (ECMO) initiation.

Group	Mortality, %	95% CI
Overall cohort	56.8	50.8–62.9
Pre-2016	58.9	44.9–73.5
Post-2016	56.4	49.9–63.1

Kaplan–Meier estimates are reported with 95% confidence intervals; patients without a documented death were censored at the date last seen. Abbreviation: CI, confidence interval.

## Data Availability

The data that support the findings of this study are not openly available due to reasons of sensitivity, but are available from the corresponding author upon reasonable request.

## Abbreviations

The following abbreviations are used in this manuscript:

VA-ECMO	Veno-arterial extracorporeal membrane oxygenation
ECMO	Extracorporeal membrane oxygenation
NIRS	Near-infrared spectroscopy
ALI	Acute limb ischemia
DPC	Distal perfusion cannula
CPR	Cardiopulmonary resuscitation
ICU	Intensive care unit
eCPR	Extracorporeal cardiopulmonary resuscitation
PAD	Peripheral arterial disease

## References

[1] Guglin M., Zucker M.J., Bazan V.M., Bozkurt B., El Banayosy A., Estep J.D., Gurley J., Nelson K., Malyala R., Panjrath G.S. (2019). Venoarterial ECMO for Adults. J. Am. Coll. Cardiol..

[2] Son A.Y., Khanh L.N., Joung H.S., Guerra A., Karim A.S., McGregor R., Pawale A., Pham D.T., Ho K.J. (2021). Limb Ischemia and Bleeding in Patients Requiring Venoarterial Extracorporeal Membrane Oxygenation. J. Vasc. Surg..

[3] Jia D., Yang I.X., Ling R.R., Syn N., Poon W.H., Murughan K., Tan C.S., Choong A.M.T.L., MacLaren G., Ramanathan K. (2020). Vascular Complications of Extracorporeal Membrane Oxygenation: A Systematic Review and Meta-Regression Analysis. Crit. Care Med..

[4] Lamb K.M., DiMuzio P.J., Johnson A., Batista P., Moudgill N., McCullough M., Eisenberg J.A., Hirose H., Cavarocchi N.C. (2017). Arterial Protocol Including Prophylactic Distal Perfusion Catheter Decreases Limb Ischemia Complications in Patients Undergoing Extracorporeal Membrane Oxygenation. J. Vasc. Surg..

[5] Lee H.-H., Jang W.J., Ahn C.-M., Chun W.J., Oh J.H., Park Y.H., Lee S.-J., Hong S.-J., Yang J.H., Kim J.-S. (2023). Association of Prophylactic Distal Perfusion Cannulation with Mortality in Patients Receiving Venoarterial Extracorporeal Membrane Oxygenation. Am. J. Cardiol..

[6] Lorusso R., Shekar K., MacLaren G., Schmidt M., Pellegrino V., Meyns B., Haft J., Vercaemst L., Pappalardo F., Bermudez C. (2021). ELSO Interim Guidelines for Venoarterial Extracorporeal Membrane Oxygenation in Adult Cardiac Patients. ASAIO J..

[7] Vinogradsky A., Kurlansky P., Ning Y., Kirschner M., Beck J., Brodie D., Kaku Y., Fried J., Takeda K. (2023). Continuous Near-Infrared Reflectance Spectroscopy Monitoring to Guide Distal Perfusion Can Minimize Limb Ischemia Surgery for Patients Requiring Femoral Venoarterial Extracorporeal Life Support. J. Vasc. Surg..

[8] Sohn B., Lee H. (2025). Near-Infrared Spectroscopy for Preventing Limb Ischemia in Extracorporeal Membrane Oxygenation. Artif. Organs.

[9] Chanan E.L., Bingham N., Smith D.E., Nunnally M.E. (2020). Early Detection, Prevention, and Management of Acute Limb Ischemia in Adults Supported with Venoarterial Extracorporeal Membrane Oxygenation. J. Cardiothorac. Vasc. Anesth..

[10] Steffen R.J., Sale S., Anandamurthy B., Cruz V.B., Grady P.M., Soltesz E.G., Moazami N. (2014). Using Near-Infrared Spectroscopy to Monitor Lower Extremities in Patients on Venoarterial Extracorporeal Membrane Oxygenation. Ann. Thorac. Surg..

[11] Kim D.J., Cho Y.-J., Park S.H., Lim C., Park K.-H., Jheon S., Kim J.S. (2017). Near-Infrared Spectroscopy Monitoring for Early Detection of Limb Ischemia in Patients on Veno-Arterial Extracorporeal Membrane Oxygenation. ASAIO J..

[12] Marbach J.A., Faugno A.J., Pacifici S., Chweich H., Marbach J.K., Rabinowitz J.B., Thayer K.L., Di Santo P., Kapur N.K. (2022). Strategies to Reduce Limb Ischemia in Peripheral Venoarterial Extracorporeal Membrane Oxygenation: A Systematic Review and Meta-Analysis. Int. J. Cardiol..

[13] Matthews R., Surti A., Bahroloomi D., Arbabi C.N., Gupta N., Baril D.T., Azizzadeh A., Gunn T.M., Chou E.L. (2026). Contemporary Practices and Limb Outcomes in Peripheral Venoarterial Extracorporeal Membrane Oxygenation at a High-Volume Single Institution. J. Vasc. Surg..

